# ZEBRA: a hierarchically integrated gene expression atlas of the murine and human brain at single-cell resolution

**DOI:** 10.1093/nar/gkad990

**Published:** 2023-11-06

**Authors:** Matthias Flotho, Jérémy Amand, Pascal Hirsch, Friederike Grandke, Tony Wyss-Coray, Andreas Keller, Fabian Kern

**Affiliations:** Helmholtz-Institute for Pharmaceutical Research Saarland (HIPS), Helmholtz Centre for Infection Research, Saarland University Campus, 66123 Saarbrücken, Germany; Clinical Bioinformatics, Center for Bioinformatics, Saarland University, 66123 Saarbrücken, Germany; Helmholtz-Institute for Pharmaceutical Research Saarland (HIPS), Helmholtz Centre for Infection Research, Saarland University Campus, 66123 Saarbrücken, Germany; Clinical Bioinformatics, Center for Bioinformatics, Saarland University, 66123 Saarbrücken, Germany; Clinical Bioinformatics, Center for Bioinformatics, Saarland University, 66123 Saarbrücken, Germany; Clinical Bioinformatics, Center for Bioinformatics, Saarland University, 66123 Saarbrücken, Germany; Department of Neurology and Neurological Sciences, Stanford University, Stanford, CA, USA; The Phil and Penny Knight Initiative for Brain Resilience, Stanford University, Stanford, CA, USA; Helmholtz-Institute for Pharmaceutical Research Saarland (HIPS), Helmholtz Centre for Infection Research, Saarland University Campus, 66123 Saarbrücken, Germany; Clinical Bioinformatics, Center for Bioinformatics, Saarland University, 66123 Saarbrücken, Germany; Helmholtz-Institute for Pharmaceutical Research Saarland (HIPS), Helmholtz Centre for Infection Research, Saarland University Campus, 66123 Saarbrücken, Germany; Clinical Bioinformatics, Center for Bioinformatics, Saarland University, 66123 Saarbrücken, Germany

## Abstract

The molecular causes and mechanisms of neurodegenerative diseases remain poorly understood. A growing number of single-cell studies have implicated various neural, glial, and immune cell subtypes to affect the mammalian central nervous system in many age-related disorders. Integrating this body of transcriptomic evidence into a comprehensive and reproducible framework poses several computational challenges. Here, we introduce ZEBRA, a large single-cell and single-nucleus RNA-seq database. ZEBRA integrates and normalizes gene expression and metadata from 33 studies, encompassing 4.2 million human and mouse brain cells sampled from 39 brain regions. It incorporates samples from patients with neurodegenerative diseases like Alzheimer’s disease, Parkinson’s disease, and Multiple sclerosis, as well as samples from relevant mouse models. We employed scVI, a deep probabilistic auto-encoder model, to integrate the samples and curated both cell and sample metadata for downstream analysis. ZEBRA allows for cell-type and disease-specific markers to be explored and compared between sample conditions and brain regions, a cell composition analysis, and gene-wise feature mappings. Our comprehensive molecular database facilitates the generation of data-driven hypotheses, enhancing our understanding of mammalian brain function during aging and disease. The data sets, along with an interactive database are freely available at https://www.ccb.uni-saarland.de/zebra.

## Graphical Abstract

**Graphical Abstract:**
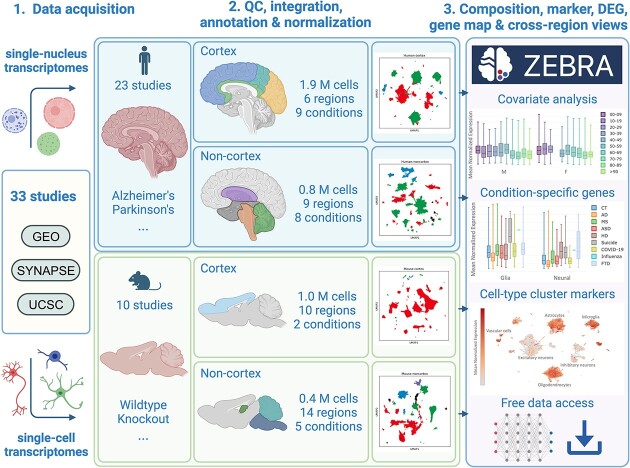

## Introduction

With demographic changes leading to a growing elderly population in Western societies, neurodegenerative diseases have received increased attention due to their direct association with aging processes, often becoming more severe in advanced age. The progression of these diseases has been linked to various genetic origins, single-nucleotide polymorphisms (SNPs), and perturbed cell-type populations (1,2). However, despite considerable progress and findings, the major molecular mechanisms underlying disease progression remain largely unknown (3). Even if it is possible to profile and classify cell-types on a fine-grained expression level, it remains difficult to interpret mechanisms and dependencies on a patient level in a comprehensive way.

Advancements in single-cell RNA-sequencing (scRNA-seq) and single-nucleus RNA-sequencing (snRNA-seq) technologies have enabled capturing of gene expression profiles at the cellular and nuclear level, respectively. This provides great insight into underlying cellular and molecular pathways linked to various pathophysiological conditions and aging processes. Although there is an exponentially growing number of freely available data sets and studies (4), the absence of a standardized nomenclature for annotation and cell labeling poses a challenge (5,6). In more detail, the data sets generated so far are still biased in two aspects. First, the choice of sample region, as mainly the cortical regions were sampled from mouse and human donors so far. Only a few studies cover multiple brain regions from the same donor. Second, the choice of sequencing technique, for human mostly single-nucleus, and in mouse samples single-cell sequencing is used. To reflect this trend, we created a scRNA-seq for the mouse and snRNA-seq atlas for the human brain accordingly.

While existing databases tailored for brain tissue and neurodegenerative diseases, such as the Allen Brain Map (7) and scREAD (8) offer valuable information based on extensive sets of scRNA-seq samples, they exhibit certain limitations. The Allen Brain Map exclusively contains studies published by the Allen Institute on the associated patient cohorts, while scREAD lacks integration of data matrices across multiple studies. In contrast to databases such as DISCO, HUSCH or HTCA which cover multiple tissues, our database is specialized in neurodegeneration and aging in the brain, covering the less frequently sampled cell-types and brain regions in much more detail (Supplementary Table S1) (9–11). Our database contains sequencing samples from 33 studies in the context of age-related and neurological disorders (Supplementary Table S2) (12–44). The distinction of brain regions is either neglected in larger databases or coarse-grained in comparison to our annotation. We recently showed that the functionally and structurally diverse regions of the mammalian brain exhibit a distinct and age-modulated transcriptome, motivating our approach to further understand the connection between molecular and cellular phenotypes in local niches (45).

All here-included studies made use of the droplet-based 10x Chromium protocol for generating libraries, leveraging the high abundance of publicly available data sets using this particular technology. By focusing on a single platform, we expect fewer technical artifacts. Only studies that provide the raw counts or SoupX-corrected counts were considered here. As a common baseline, we applied doublet removal and filtering with carefully selected thresholds to ensure the quality of the included cells and nuclei. To integrate hundreds of samples effectively and efficiently, we employ the generative and deep probabilistic auto-encoder model scVI (46). Using a training procedure, we generated a latent space representation based on the posterior distribution of the gene counts. We only use the resulting latent space representation for clustering and visualization.

ZEBRA is the first large-scale database enabling an overview and gene-wise analysis of scRNA-seq / snRNA-seq samples across diverse studies while preserving the details on cell-type and regional annotation. Moreover, ZEBRA is a valuable resource for easy-to-access gene analysis functionality in the context of aging and neurodegeneration. We enable robust analysis in cortical as well as non-cortical brain regions. Finally, our human cortex data set is the first of its kind integrating and providing human brain cell transcriptomes across almost the entire human age, i.e. from early childhood to late adulthood.

## Materials and methods

### Data collection

The data was collected from Gene Expression Omnibus (GEO) (47), Synapse and the UCSC Cell Browser (48). Only count and SoupX-corrected count matrices (49) were used for our database. The considered samples were exclusively generated using the droplet-based 10x Chromium 3′ gene expression protocol (50). In particular, we only include human single-nucleus and mouse single-cell RNA sequencing studies. We explicitly exclude studies related to embryonic development and cancer progression due to their nature of inducing very high transcriptomic variability. We also exclude studies with human data that do not allow unique donor mappings from cell to donor (51). Furthermore, only studies that used the GRCh38 human genome or the GRCm38 mouse genome as a reference were considered. The metadata was manually curated, standardized, and checked. The cell-type annotation was performed manually, i.e. similar cell-types have been mapped onto each other and redundancies have been removed. Subsequently, we re-annotated the cell-types to fill in missing or to correct annotations. We provide a continuous and categorical scale about the age information across human samples and categorical information for the mouse samples. The sexes were summarized into male (M), female (F), undefined and mixed. ‘Mixed’ describes mouse samples where multiple sexes have been pooled together. Finally, we summarized the information about medical conditions into super-groups merging MS and AD sub-types, respectively. The processed, re-annotated and integrated data can be downloaded from the server. Original raw and normalized counts are also available. Data sets with access restrictions due to sensitive patient data are removed from the downloadable data files (16,17,24,31).

### Preprocessing

We used the Scanpy package (v1.9.2 with Anndata v0.8.0) for preprocessing as a wrapper for the expression data. For each study, we applied Scrublet (v0.2.3) for detecting and removing putative doublets. The different data sets were merged by mapping equivalent genes onto each other and appending the observations to a single joint expression matrix. Gene isoforms were summarized by a single gene label, i.e., the counts over all isoforms were summed up to a single gene label. To include a gene in the atlas it must be present in at least half of the data sets. Based on this gene set, we also create and provide the count matrix containing the isoforms but exclude it from our downstream analysis. Missing gene entries have been treated as NaN and have not been considered for DEG computation or integration. Cells were filtered out if they contained >5% mitochondrial counts (52), >7500 genes per cell, or <200 genes. Lastly, genes detected in less than 3 cells have been removed from the atlas. We then normalized and scaled the single-cell and single-nucleus count matrices according to the Scanpy workflow using the sc.pp.normalize_total and sc.pp.log1p functions.

### Integration

ScVI (v0.17.3 with PyTorch v1.12.1) was used for integrating the preprocessed counts on a NVIDIA A100 GPU machine. As input, the count matrix is reduced to genes that are present in all data sets. This allows to compute normalized scVI counts for the largest possible number of genes in the database. ScVI was executed using default parameters and 1000 epochs at max. An epoch is defined as the cycle in which the model is trained on the entire training data exactly once. In each epoch the weights update until the maximum of epochs is reached or there is no significant change in model performance. For all sub-data sets the training converged before reaching 1000 training epochs. The integration was performed on the sub-data sets split both by brain region (cortex and non-cortex) and by species.

### Curated cell-type annotation

We summarized existing annotations to a two-level cell-type hierarchy, harmonizing the cell-type annotations from the collected studies. We then re-annotated the cortex cell-types based on the integrated representation. Here, we clustered the cells using the RAPIDS cuGraph Leiden algorithm implementation and the RAPIDS cuml umap (v22.06.01) (53) to identify and define cell-type clusters. The cluster names have been derived from examining the majority of original cell-type present in each cluster and by the marker genes reported in several of the included studies (12,19,30).

### Differentially expressed gene (DEG) computation

For computing DEG statistics we used the edgeR (v3.36.0) Bioconductor package on aggregated pseudo-bulk samples. We aggregated by summing up the counts of cells split by ‘donor’, ‘region2’ and ‘sub_cell_type’ labels. The pseudo-bulk samples were processed and normalized according to the edgeR tutorial using the glmQLFTest function. Whenever we computed the DEGs or markers between more than one study, we included the ‘study’ information as latent-variable in the model design matrix. We provide cell-type markers computed for each cluster against all other cells and putative marker genes for the included diseases. The disease markers have been computed for each cell-type distinctively, i.e., the conditions were compared within the same cell-type but not across multiple cell-types. Additionally, we provide the pairwise DEGs of all cell-types across regions within the same species in separate views. Cell expression vectors containing NaN entries for certain genes have not been used for computing the DEGs. We used the stats R (v4.1.3) package for adjusting the p-values for the condition markers, with the p.adjust method set to use the Benjamini-Hochberg adjustment false-discovery rate (‘BH’) procedure. All computed markers and DEGs can be downloaded from the server.

### Database implementation

We implemented an online database that allows the user to download the atlas data, explore the data set composition and visualize gene expression across cells. The database is implemented using the latest Python (v3.11) and Django (v4.2) framework releases in a reproducible Docker setup. The front end uses Bootstrap (v5.2), DataTables (v1.13) and the Plotly.js (v2.25) plotting library. The database is freely available at: https://www.ccb.uni-saarland.de/zebra/.

## Results

### Overview

**Figure 1.**
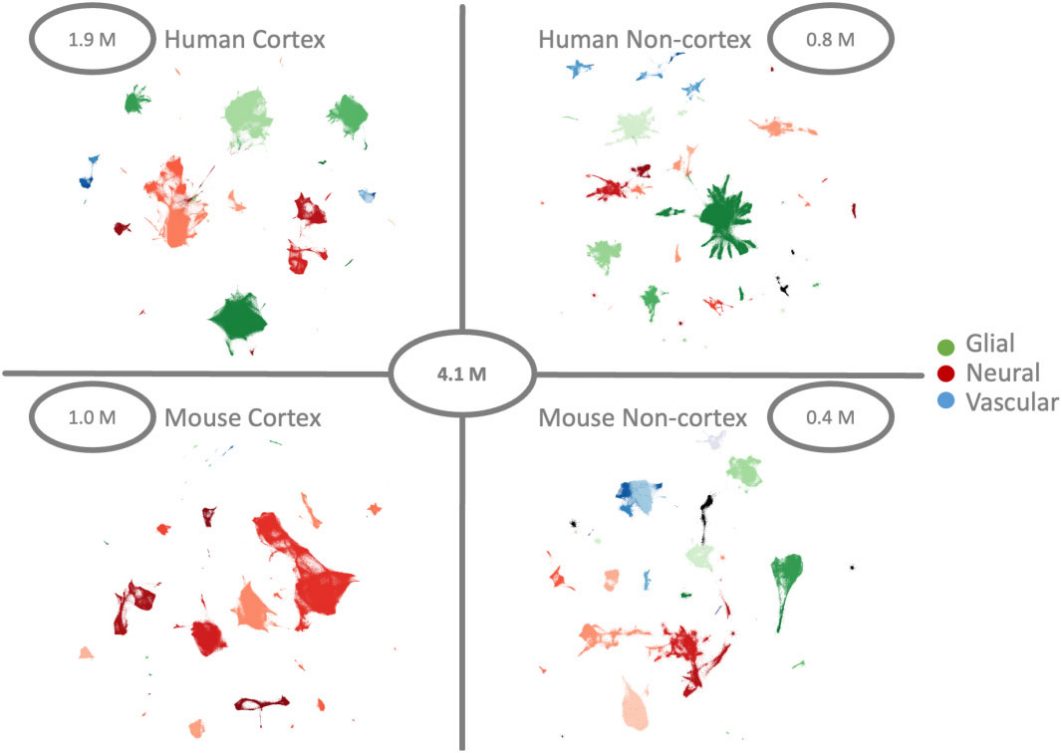
The ZEBRA brain atlas contains a total of 4.1 million human and mouse brain cells and nuclei. These are split into two larger cortex data sets and two smaller non-cortex data sets. For each data set, the cellular and nuclear transcriptomes are preprocessed and embedded into a UMAP, colored by cell-type lineage. The main cell populations are glial (greens), neural (reds) and vascular (blues) cells. The number of cells or nuclei per main data set is shown in each subplot.

Our database includes 33 studies with 2 743 355 human and 1 414 605 mouse cells. We use a hierarchical approach to organize the data based on the sample region (Figure 1). The human data set splits into 1 930 270 cortical and 813 085 non-cortical nuclei. The mouse cortex samples include 1 000 166 cells and 414 439 cells from outside the cortex. Diverse levels of data integration showed that the best integration results were achieved when separating cortex and non-cortex samples. The sampled regions are sometimes only captured in a single study. Moreover, the overlaps of cell-types across locations are often small. In general, the collected studies are heterogeneous on several levels: the sequencing depth is different, the cell-type annotation is inconsistent, and the sample locations vary.

### Data set description

We removed redundancies and curated the original cell-types by merging them into unique labels structured into two levels. To this end, we re-annotate all cells into coarser super- and finer sub-cell-types to improve consistency across all studies. Besides, we unify the annotation of the batches, sampling region, age, sex, and medical condition. The collected human samples include donors of a variety of different diseases (Table 1). For example, our atlas includes samples from 196 distinct human control donors and 88 donors with Alzheimer’s disease. We observe slightly more male than female samples and cells. In the mouse atlas, we report mostly wild-type (WT) samples with 204 unique donor labels, where 82 labels correspond to mixed sexes, meaning that at least 2 individuals have been pooled together. The integrated representation has large overlaps between assigned cell-types and predicted Leiden clusters. Additionally, we manually curated the cell-type annotation of the cortex and non-cortex samples. Our improved annotation aims to preserve the granularity of the provided cluster labels while improving cell-type classification. In contrast to the favorable integration results in cortex samples, the model training process across non-cortex regions was challenging due to certain brain regions being selectively covered by a single study. To further investigate these region-driven differences we integrated samples from each super-cell-type present in multiple regions separately. The subsequent result shows that the cells indeed cluster according to their expected sub-cell-types. Therefore, the brain-region-driven effects that made integrating the cross-region samples hard, could be minimized by preselecting populations of similar cells while avoiding the risk to remove valuable biological signals from a combination of transcriptionally distinct brain regions.

**Table 1.** The number of donors varies across different medical conditions and covariates

Species	Condition	#Donors	#Cells	M/F/mixed/
				unknown
Human	CT	196	1301k	128/69/0/1
	AD	88	529k	48/40/0/0
	ASD	21	148k	17/4/0/0
	COVID-19	8	33k	7/1/0/0
	FTD	27	251k	11/16/0/0
	HD	12	87k	9/3/0/0
	Influenza	1	5k	1/0/0/0
	LBD	4	61k	2/2/0/0
	MS	30	159k	24/8/0/1
	PD	6	123k	4/2/0/0
	Suicide	17	43k	17/0/0/0
Mouse	WT	204	1361k	61/48/82/15
	EAE	3	23k	0/0/0/3
	MCAO	3	26k	0/0/0/3
	MA	2	4k	0/2/0/0
	hGFAP-GFP	1	1k	0/0/1/0

While the metadata label for the sex is mostly present for the human donors, we observe more unlabeled or mixed donors in the mouse models. We collected samples from Alzheimer’s disease (AD), autism spectrum disorder (ASD), SARS-CoV-2 (COVID-19), frontotemporal dementia (FTD), Huntington’s disease (HD), influenza, Lewy body dementia (LBD), Multiple Sclerosis (MS), Parkinson’s disease (PD), and depressive disorder (Suicide) patients. Besides, ZEBRA contains samples from wild-type (WT), experimental autoimmune encephalomyelitis (EAE), microglia absence (MA), and fluorescent astrocytes (hGFAP-GFP) mice.

### Database functionality

ZEBRA is an interactive database that provides a comprehensive cross-study overview of the human and mouse brain in aging and neurodegenerative diseases. It gives access to the key-findings without downloading the complete data set. Each view is designed to answer a series of questions based on pre-calculated analyses. One core functionality of the web page is to visualize the UMAP embedding of each of the four main data sets based on metadata like cell-type or the expression of genes of interest. All plots are interactive, allowing zooming, downloading, and toggling of the visibility of categories. In addition, the embedding view can be sliced by the available metadata variables. The user can analyze the data set composition by comparing experimental factors to each other. For example, the proportions of cells from selected conditions across each cell-type may be plotted to ensure that the data set is balanced subject to specific downstream analyses.

Additionally, we provide the results of a differential gene expression analysis for each gene in the data set. The database allows the visualization of the mean gene expression on the single-cell and single-nucleus level grouped by categories such as cell-type, original data set, or sex. Both, the embedding and the DEG analysis can be used to readily look up marker genes for the purpose of annotating new data sets. The DEGs can be easily filtered by sample number, e.g. the gene has to be present in at least *n* samples in each condition, as to enforce statistical stringency. Moreover, ZEBRA enables an easy way to compare homologous genes between human and mouse brain regions. The integrated data sets can be downloaded as H5AD objects for use with Scanpy. Finally, ZEBRA provides pairwise DEGs between each cell type both across major brain regions and within the same region. This enables a detailed view of how similar cell-types differ in their transcriptome across the brain but also which DEGs are distinct between related cell-type lineages.

### Exemplified use-cases

**Figure 2.**
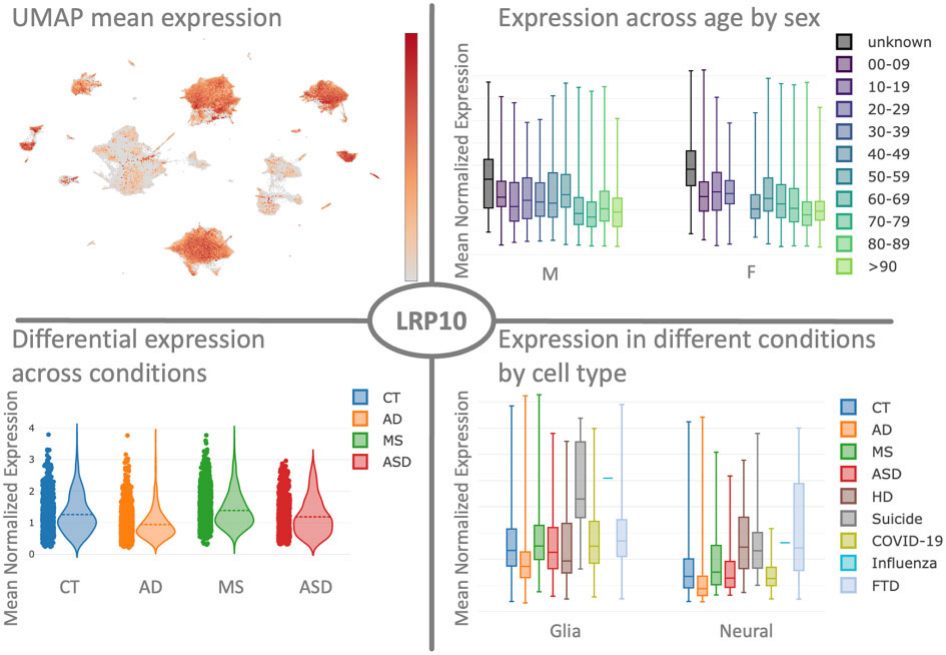
Case study examining the gene LRP10 in human cortex: ZEBRA provides a series of useful and readily accessible views to examine genes of interest. From top left to bottom right: the mean expression of selected genes can be plotted in a UMAP projection to detect cell-type specific expression patterns (Embedding view). The mean gene expression can also be grouped by factors of interest such as age or sex to find trends of association (Gene Map view). ZEBRA provides a view of the DEG analysis for each gene (Diff. Expression view). The Gene Map view finally allows combining several categories at once, for instance, all conditions per cell-type.

In Figure 2, possible use-cases and views of the ZEBRA database for the gene LRP10 in the human cortex are shown. It is a known key-driver for sex-specific networks in AD (54). Using ZEBRA we can easily visualize how the expression varies in different cell-types using the embedding view, and how it is expressed in males versus females by age. In particular, due to the age range covered in our human data, it is easy to compare changes in gene expression for different conditions and sexes via the gene map view and across a broad age range. The database tools support independent validation procedures or aids in finding other physiological conditions that can affect a gene in certain cell-types. Besides, ZEBRA can in principle be used to train and benchmark new or existing cell type prediction tools. Automatically labelling cells based on their transcriptomic signature is an on-going scientific challenge for which sufficiently sized and broadly covered reference databases are urgently required (55).

## Discussion

The landscape of freely available scRNA-seq and snRNA-seq studies on the mammalian brain continues to expand. However, the absence of universal nomenclatures and minimal but standardized requirements for published data remains a challenge in the field. Consequently, integrating and comparing all available information proves problematic, necessitating extensive manual curation. Furthermore, the compatibility between a plethora of existing frameworks for handling and storing scRNA-seq data (loompy: http://linnarssonlab.org/loompy, SeuratDisk: https://mojaveazure.github.io/seurat-disk, scvi-tools (56)) is poor and subject to on-going breaking changes that hinder the accessibility for non-computational research experts to perform cross-study comparisons or analyses of reproducibility.

In this study, we introduced ZEBRA, a database that provides access to 33 manually curated and integrated scRNA-seq and snRNA-seq studies. To this end, we combined the cellular and nuclear transcriptomes on several levels to create a hierarchical design of our database. Recognizing that cross-species integration is difficult due to differing genomic annotations and gene functions as well as the fact that single-cell sequencing is more frequently performed for mouse than for human, and vice-versa for single-nucleus sequencing, a split by species is required to alleviate the need for extensive technical batch effect correction. Moreover, we observed that for both human and mouse the number of samples available per brain-region is heavily biased towards cortex, for which we can identify multiple reasons. This approach showed best-performing integration results as it balances statistical power and sensitivity of picking up pronounced cell-type differences in gene expression for the well-covered cortex and less covered non-cortical regions. Consequently, our results suggest that future endeavors should consider sampling across multiple regions within a single individual to enhance computational integration.

By clustering integrated human and mouse cortex samples, we observe a substantial overlap between the computationally derived clusters and the original cell-type annotations in a majority-vote manner. The re-annotated cells in ZEBRA provide more consistent cell-type labeling. For example, we could observe inconsistencies in how OPCs and oligodendrocytes were previously labeled across different studies. ZEBRA offers a reliable reference for human brain marker genes, as we confirmed most of the annotated cells while relabeling mislabeled cells. Additionally, our findings highlight the general reproducibility of droplet-based scRNA-seq and snRNA-seq protocols, as we successfully integrated human cortical cells from 19 distinct studies. The presented data shows the heterogeneity across different brain regions, emphasizing the critical role of the spatial locality as a driving factor in cellular diversity (45). Distinguishing between batch effects and biological signals proves challenging, as most studies sample only one single location of the brain. Here we recognized also the diversity of tissue homogenization and cell extraction protocols currently reported among the single-cell literature, each leading to individual biases and noise that is challenging to regress out computationally, especially when sample numbers are low. Settling on common and approved standards could certainly help to improve the overall reproducibility of single-cell research, especially in clinical and drug development contexts.

Future work for ZEBRA could comprise a complete realignment of all raw reads to further improve the overall data quality and to better resolve transcriptional isoforms. Still, the 3′ gene expression technology used by most studies unevenly covers gene transcripts, with most reads aligning to a region close the 3′ UTR. This makes a consistent detection of splicing events inherently difficult to measure (57). Alternative full-length but more labor-intensive platforms such as Smart-seq2 have already been established but are adopted more slowly (58). Comprehensively integrating full-length and 3′ droplet-based counts is then another computational challenge to be resolved should more full-length data become available over time. Nevertheless we account for this use-case by reporting also the original isoform counts for each study, where available.

The provided database serves as a new reference for forthcoming experiments and to guide cohort design, facilitating also complex computational tasks such as cell-type annotation and benchmarking of novel cell-type prediction tools. Such an extensive compilation of data sets enables a more robust evaluation of cellular and nuclear transcriptomes at scale and with ease. We hope that ZEBRA will be a valuable resource for neurodegenerative disease and aging research, fostering the rapid development of novel therapeutic approaches.

## Supplementary Material

gkad990_Supplemental_Files

## Data Availability

ZEBRA is freely available at https://www.ccb.uni-saarland.de/zebra.

## References

[1] Brandebura A.N. , Paumier A., Onur T.S., Allen N.J. Astrocyte contribution to dysfunction, risk and progression in neurodegenerative disorders. Nat. Rev. Neurosci. 2023; 24:23–39. 36316501 10.1038/s41583-022-00641-1 PMC10198620

[2] Klein C. , Westenberger A. Genetics of Parkinson’s disease. Cold Spring Harbor Perspect. Med. 2012; 2:a008888. 10.1101/cshperspect.a008888 PMC3253033 22315721

[3] Barnes J. , Dickerson B.C., Frost C., Jiskoot L.C., Wolk D., Flier W.M. Alzheimer’s disease first symptoms are age dependent: evidence from the NACC dataset. Alzheimer Dement. 2015; 11:1349–1357. 10.1016/j.jalz.2014.12.007 PMC4619185 25916562

[4] Svensson V. , Vento-Tormo R., Teichmann S.A. Exponential scaling of single-cell RNA-seq in the past decade. Nat. Protoc. 2018; 13:599–604. 29494575 10.1038/nprot.2017.149

[5] Miller J.A. , Gouwens N.W., Tasic B., Collman F., van Velthoven C.T., Bakken T.E., Hawrylycz M.J., Zeng H., Lein E.S., Bernard A. Common cell type nomenclature for the mammalian brain. eLife. 2020; 9:e59928. 33372656 10.7554/eLife.59928 PMC7790494

[6] Zeng H. What is a cell type and how to define it?. Cell. 2022; 185:2739–2755. 35868277 10.1016/j.cell.2022.06.031 PMC9342916

[7] Gabitto M.I. , Travaglini K.J., Rachleff V.M., Kaplan E.S., Long B., Ariza J., Ding Y., Mahoney J.T., Dee N., Goldy J. et al. Integrated multimodal cell atlas of Alzheimer’s disease. 2023; bioRxiv doi:09 May 2023, preprint: not peer reviewed https://www.biorxiv.org/content/10.1101/2023.05.08.539485v1. 10.1038/s41593-024-01774-5 PMC11614693 39402379

[8] Jiang J. , Wang C., Qi R., Fu H., Ma Q. scREAD: a single-cell RNA-seq database for Alzheimer’s disease. iScience. 2020; 23:101769. 33241205 10.1016/j.isci.2020.101769 PMC7674513

[9] Pan L. , Shan S., Tremmel R., Li W., Liao Z., Shi H., Chen Q., Zhang X., Li X. HTCA: a database with an in-depth characterization of the single-cell human transcriptome. Nucleic Acids Res. 2022; 51:D1019–D1028. 10.1093/nar/gkac791 PMC9825435 36130266

[10] Shi X. , Yu Z., Ren P., Dong X., Ding X., Song J., Zhang J., Li T., Wang C. HUSCH: an integrated single-cell transcriptome atlas for human tissue gene expression visualization and analyses. Nucleic Acids Res. 2022; 51:D1029–D1037. 10.1093/nar/gkac1001 PMC9825509 36318258

[11] Li M. , Zhang X., Ang K.S., Ling J., Sethi R., Lee N., Ginhoux F., Chen J. DISCO: a database of Deeply Integrated human Single-Cell Omics data. Nucleic Acids Res. 2021; 50:D596–D602. 10.1093/nar/gkab1020 PMC8728243 34791375

[12] Yao Z. , van Velthoven C.T., Nguyen T.N., Goldy J., Sedeno-Cortes A.E., Baftizadeh F., Bertagnolli D., Casper T., Chiang M., Crichton K. et al. A taxonomy of transcriptomic cell types across the isocortex and hippocampal formation. Cell. 2021; 184:3222–3241. 34004146 10.1016/j.cell.2021.04.021 PMC8195859

[13] Kamath T. , Abdulraouf A., Burris S.J., Langlieb J., Gazestani V., Nadaf N.M., Balderrama K., Vanderburg C., Macosko E.Z. Single-cell genomic profiling of human dopamine neurons identifies a population that selectively degenerates in Parkinson’s disease. Nat. Neurosci. 2022; 25:588–595. 35513515 10.1038/s41593-022-01061-1 PMC9076534

[14] Gerrits E. , Giannini L.A.A., Brouwer N., Melhem S., Seilhean D., Ber I.L., Kamermans A., Kooij G., de Vries H.E., Boddeke E.W.G.M. et al. Neurovascular dysfunction in GRN-associated frontotemporal dementia identified by single-nucleus RNA sequencing of human cerebral cortex. Nat. Neurosci. 2022; 25:1034–1048. 35879464 10.1038/s41593-022-01124-3

[15] Sayed F.A. , Kodama L., Fan L., Carling G.K., Udeochu J.C., Le D., Li Q., Zhou L., Wong M.Y., Horowitz R. et al. AD-linked R47H- TREM mutation induces disease-enhancing microglial states via AKT hyperactivation. Sci. Transl. Med. 2021; 13:eabe3947. 34851693 10.1126/scitranslmed.abe3947 PMC9345574

[16] Gandal M.J. , Haney J.R., Wamsley B., Yap C.X., Parhami S., Emani P.S., Chang N., Chen G.T., Hoftman G.D., de Alba D. et al. Broad transcriptomic dysregulation occurs across the cerebral cortex in ASD. Nature. 2022; 611:532–539. 36323788 10.1038/s41586-022-05377-7 PMC9668748

[17] Blanchard J.W. , Akay L.A., Davila-Velderrain J., von Maydell D., Mathys H., Davidson S.M., Effenberger A., Chen C.-Y., Maner-Smith K., Hajjar I. et al. APOE4 impairs myelination via cholesterol dysregulation in oligodendrocytes. Nature. 2022; 611:769–779. 36385529 10.1038/s41586-022-05439-w PMC9870060

[18] Zeisel A. , Hochgerner H., Lönnerberg P., Johnsson A., Memic F., van der Zwan J., Häring M., Braun E., Borm L.E., Manno G.L. et al. Molecular architecture of the mouse nervous system. Cell. 2018; 174:999–1014. 30096314 10.1016/j.cell.2018.06.021 PMC6086934

[19] Yang A.C. , Vest R.T., Kern F., Lee D.P., Agam M., Maat C.A., Losada P.M., Chen M.B., Schaum N., Khoury N. et al. A human brain vascular atlas reveals diverse mediators of Alzheimer’s risk. Nature. 2022; 603:885–892. 35165441 10.1038/s41586-021-04369-3 PMC9635042

[20] Ayhan F. , Kulkarni A., Berto S., Sivaprakasam K., Douglas C., Lega B.C., Konopka G. Resolving cellular and molecular diversity along the hippocampal anterior-to-posterior axis in humans. Neuron. 2021; 109:2091–2105. 34051145 10.1016/j.neuron.2021.05.003 PMC8273123

[21] Herring C.A. , Simmons R.K., Freytag S., Poppe D., Moffet J.J., Pflueger J., Buckberry S., Vargas-Landin D.B., Clément O., Echeverría E.G. et al. Human prefrontal cortex gene regulatory dynamics from gestation to adulthood at single-cell resolution. Cell. 2022; 185:4428–4447. 36318921 10.1016/j.cell.2022.09.039

[22] Velmeshev D. , Schirmer L., Jung D., Haeussler M., Perez Y., Mayer S., Bhaduri A., Goyal N., Rowitch D.H., Kriegstein A.R. Single-cell genomics identifies cell type–specific molecular changes in autism. Science. 2019; 364:685–689. 31097668 10.1126/science.aav8130 PMC7678724

[23] Garcia F.J. , Sun N., Lee H., Godlewski B., Mathys H., Galani K., Zhou B., Jiang X., Ng A.P., Mantero J. et al. Single-cell dissection of the human brain vasculature. Nature. 2022; 603:893–899. 35158371 10.1038/s41586-022-04521-7 PMC9680899

[24] Mathys H. , Davila-Velderrain J., Peng Z., Gao F., Mohammadi S., Young J.Z., Menon M., He L., Abdurrob F., Jiang X. et al. Single-cell transcriptomic analysis of Alzheimer’s disease. Nature. 2019; 570:332–337. 31042697 10.1038/s41586-019-1195-2 PMC6865822

[25] Lim R.G. , Al-Dalahmah O., Wu J., Gold M.P., Reidling J.C., Tang G., Adam M., Dansu D.K., Park H.-J., Casaccia P. et al. Huntington disease oligodendrocyte maturation deficits revealed by single-nucleus RNAseq are rescued by thiamine-biotin supplementation. Nat. Commun. 2022; 13:7791. 36543778 10.1038/s41467-022-35388-x PMC9772349

[26] Nagy C. , Maitra M., Tanti A., Suderman M., Théroux J.-F., Davoli M.A., Perlman K., Yerko V., Wang Y.C., Tripathy S.J. et al. Single-nucleus transcriptomics of the prefrontal cortex in major depressive disorder implicates oligodendrocyte precursor cells and excitatory neurons. Nat. Neurosci. 2020; 23:771–781. 32341540 10.1038/s41593-020-0621-y

[27] BRAIN Initiative Cell Census Network (BICCN) Callaway E.M. , Ascoli G.A., Huang Z.J. A multimodal cell census and atlas of the mammalian primary motor cortex. Nature. 2021; 598:86–102. 34616075 10.1038/s41586-021-03950-0 PMC8494634

[28] Zhao L. , Li Z., Vong J.S.L., Chen X., Lai H.-M., Yan L.Y.C., Huang J., Sy S.K.H., Tian X., Huang Y. et al. Pharmacologically reversible zonation-dependent endothelial cell transcriptomic changes with neurodegenerative disease associations in the aged brain. Nat. Commun. 2020; 11:4413. 32887883 10.1038/s41467-020-18249-3 PMC7474063

[29] Absinta M. , Maric D., Gharagozloo M., Garton T., Smith M.D., Jin J., Fitzgerald K.C., Song A., Liu P., Lin J.-P. et al. A lymphocyte–microglia–astrocyte axis in chronic active multiple sclerosis. Nature. 2021; 597:709–714. 34497421 10.1038/s41586-021-03892-7 PMC8719282

[30] Yang A.C. , Kern F., Losada P.M., Agam M.R., Maat C.A., Schmartz G.P., Fehlmann T., Stein J.A., Schaum N., Lee D.P. et al. Dysregulation of brain and choroid plexus cell types in severe COVID-19. Nature. 2021; 595:565–571. 34153974 10.1038/s41586-021-03710-0 PMC8400927

[31] Morabito S. , Miyoshi E., Michael N., Shahin S., Martini A.C., Head E., Silva J., Leavy K., Perez-Rosendahl M., Swarup V. Single-nucleus chromatin accessibility and transcriptomic characterization of Alzheimer’s disease. Nat. Genet. 2021; 53:1143–1155. 34239132 10.1038/s41588-021-00894-z PMC8766217

[32] Zheng K. , Lin L., Jiang W., Chen L., Zhang X., Zhang Q., Ren Y., Hao J. Single-cell RNA-seq reveals the transcriptional landscape in ischemic stroke. J. Cerebral Blood Flow Metab. 2021; 42:56–73. 10.1177/0271678X211026770 PMC8721774 34496660

[33] Fournier A.P. , Tastet O., Charabati M., Hoornaert C., Bourbonnière L., Klement W., Larouche S., Tea F., Wang Y.C., Larochelle C. et al. Single-Cell Transcriptomics Identifies Brain Endothelium Inflammatory Networks in Experimental Autoimmune Encephalomyelitis. Neurol. Neuroimmunol. Neuroinflam. 2022; 10:e200046. 10.1212/NXI.0000000000200046 PMC9709715 36446612

[34] Schirmer L. , Velmeshev D., Holmqvist S., Kaufmann M., Werneburg S., Jung D., Vistnes S., Stockley J.H., Young A., Steindel M. et al. Neuronal vulnerability and multilineage diversity in multiple sclerosis. Nature. 2019; 573:75–82. 31316211 10.1038/s41586-019-1404-z PMC6731122

[35] Trobisch T. , Zulji A., Stevens N.A., Schwarz S., Wischnewski S., Öztürk M., Perales-Patón J., Haeussler M., Saez-Rodriguez J., Velmeshev D. et al. Cross-regional homeostatic and reactive glial signatures in multiple sclerosis. Acta Neuropathol. 2022; 144:987–1003. 36112223 10.1007/s00401-022-02497-2 PMC9547805

[36] Kihara Y. , Zhu Y., Jonnalagadda D., Romanow W., Palmer C., Siddoway B., Rivera R., Dutta R., Trapp B.D., Chun J. Single-nucleus RNA-seq of normal-appearing brain regions in relapsing-remitting vs. secondary progressive multiple sclerosis: implications for the efficacy of fingolimod. Front. Cell. Neurosci. 2022; 16:918041. 35783097 10.3389/fncel.2022.918041 PMC9247150

[37] Durante M.A. , Kurtenbach S., Sargi Z.B., Harbour J.W., Choi R., Kurtenbach S., Goss G.M., Matsunami H., Goldstein B.J. Single-cell analysis of olfactory neurogenesis and differentiation in adult humans. Nat. Neurosci. 2020; 23:323–326. 32066986 10.1038/s41593-020-0587-9 PMC7065961

[38] Hochgerner H. , Zeisel A., Lönnerberg P., Linnarsson S. Conserved properties of dentate gyrus neurogenesis across postnatal development revealed by single-cell RNA sequencing. Nat. Neurosci. 2018; 21:290–299. 29335606 10.1038/s41593-017-0056-2

[39] Jäkel S. , Agirre E., Falcão A.M., van Bruggen D., Lee K.W., Knuesel I., Malhotra D., ffrench Constant C., Williams A., Castelo-Branco G. Altered human oligodendrocyte heterogeneity in multiple sclerosis. Nature. 2019; 566:543–547. 30747918 10.1038/s41586-019-0903-2 PMC6544546

[40] Hardwick S.A. , Hu W., Joglekar A., Fan L., Collier P.G., Foord C., Balacco J., Lanjewar S., Sampson M.M., Koopmans F. et al. Single-nuclei isoform RNA sequencing unlocks barcoded exon connectivity in frozen brain tissue. Nat. Biotechnol. 2022; 40:1082–1092. 35256815 10.1038/s41587-022-01231-3 PMC9287170

[41] Dulken B.W. , Buckley M.T., Negredo P.N., Saligrama N., Cayrol R., Leeman D.S., George B.M., Boutet S.C., Hebestreit K., Pluvinage J.V. et al. Single-cell analysis reveals T cell infiltration in old neurogenic niches. Nature. 2019; 571:205–210. 31270459 10.1038/s41586-019-1362-5 PMC7111535

[42] McNamara N.B. , Munro D.A.D., Bestard-Cuche N., Uyeda A., Bogie J. F.J., Hoffmann A., Holloway R.K., Molina-Gonzalez I., Askew K.E., Mitchell S. et al. Microglia regulate central nervous system myelin growth and integrity. Nature. 2022; 613:120–129. 36517604 10.1038/s41586-022-05534-y PMC9812791

[43] Parker K.R. , Migliorini D., Perkey E., Yost K.E., Bhaduri A., Bagga P., Haris M., Wilson N.E., Liu F., Gabunia K. et al. Single-Cell analyses identify brain mural cells expressing CD19 as potential off-tumor targets for CAR-T immunotherapies. Cell. 2020; 183:126–142. 32961131 10.1016/j.cell.2020.08.022 PMC7640763

[44] Mathew A.S. , Gorick C.M., Price R.J. Single-cell mapping of focused ultrasound-transfected brain. Gene Ther. 2021; 30:255–263. 33526842 10.1038/s41434-021-00226-0 PMC8325700

[45] Hahn O. , Foltz A.G., Atkins M., Kedir B., Moran-Losada P., Guldner I.H., Munson C., Kern F., Pálovics R., Lu N. et al. Atlas of the aging mouse brain reveals white matter as vulnerable foci. Cell. 2023; 186:4117–4133. 37591239 10.1016/j.cell.2023.07.027 PMC10528304

[46] Lopez R. , Regier J., Cole M.B., Jordan M.I., Yosef N. Deep generative modeling for single-cell transcriptomics. Nat. Methods. 2018; 15:1053–1058. 30504886 10.1038/s41592-018-0229-2 PMC6289068

[47] Barrett T. , Wilhite S.E., Ledoux P., Evangelista C., Kim I.F., Tomashevsky M., Marshall K.A., Phillippy K.H., Sherman P.M., Holko M. et al. NCBI GEO: archive for functional genomics data sets–update. Nucleic Acids Res. 2012; 41:D991–D995. 23193258 10.1093/nar/gks1193 PMC3531084

[48] Speir M.L. , Bhaduri A., Markov N.S., Moreno P., Nowakowski T.J., Papatheodorou I., Pollen A.A., Raney B.J., Seninge L., Kent W.J., et al. UCSC cell browser: visualize your single-cell data. Bioinformatics. 2021; 37:4578–4580. 34244710 10.1093/bioinformatics/btab503 PMC8652023

[49] Young M.D. , Behjati S. SoupX removes ambient RNA contamination from droplet-based single-cell RNA sequencing data. GigaScience. 2020; 9:giaa151. 33367645 10.1093/gigascience/giaa151 PMC7763177

[50] Zheng G.X. , Terry J.M., Belgrader P., Ryvkin P., Bent Z.W., Wilson R., Ziraldo S.B., Wheeler T.D., McDermott G.P., Zhu J. et al. Massively parallel digital transcriptional profiling of single cells. Nat. Commun. 2017; 8:14049. 28091601 10.1038/ncomms14049 PMC5241818

[51] Grubman A. , Chew G., Ouyang J.F., Sun G., Choo X.Y., McLean C., Simmons R.K., Buckberry S., Vargas-Landin D.B., Poppe D. et al. A single-cell atlas of entorhinal cortex from individuals with Alzheimer’s disease reveals cell-type-specific gene expression regulation. Nat. Neurosci. 2019; 22:2087–2097. 31768052 10.1038/s41593-019-0539-4

[52] Osorio D. , Cai J.J. Systematic determination of the mitochondrial proportion in human and mice tissues for single-cell RNA-sequencing data quality control. Bioinformatics. 2021; 37:963–967. 32840568 10.1093/bioinformatics/btaa751 PMC8599307

[53] Nolet C. , Lal A., Ilango R., Dyer T., Movva R., Zedlewski J., Israeli J. Accelerating single-cell genomic analysis with GPUs. 2022; bioRxiv doi:28 May 2022, preprint: not peer reviewed 10.1101/2022.05.26.493607.

[54] Guo L. , Cao J., Hou J., Li Y., Huang M., Zhu L., Zhang L., Lee Y., Duarte M.L., Zhou X. et al. Sex specific molecular networks and key drivers of Alzheimer’s disease. Mol. Neurodegener. 2023; 18:1–25. 37340466 10.1186/s13024-023-00624-5 PMC10280841

[55] Lähnemann D. , Köster J., Szczurek E., McCarthy D.J., Hicks S.C., Robinson M.D., Vallejos C.A., Campbell K.R., Beerenwinkel N., Mahfouz A. et al. Eleven grand challenges in single-cell data science. Genome Biol. 2020; 21:31. 32033589 10.1186/s13059-020-1926-6 PMC7007675

[56] Gayoso A. , Lopez R., Xing G., Boyeau P., Valiollah Pour Amiri V., Hong J., Wu K., Jayasuriya M., Mehlman E., Langevin M. et al. A python library for probabilistic analysis of single-cell omics data. Nat. Biotechnol. 2022; 40:163–166. 35132262 10.1038/s41587-021-01206-w

[57] Arzalluz-Luque A. , Conesa A. Single-cell RNAseq for the study of isoforms—how is that possible. Genome Biol. 2018; 19:110. 30097058 10.1186/s13059-018-1496-z PMC6085759

[58] Picelli S. , Faridani O.R., Björklund A.K., Winberg G., Sagasser S., Sandberg R. Full-length RNA-seq from single cells using Smart-seq2. Nat. Protoc. 2014; 9:171–181. 24385147 10.1038/nprot.2014.006

