# Supplementary material for: ZEBRA: a hierarchically integrated gene expression atlas of the murine and human brain at single-cell resolution
Source: Nucleic Acids Res. 2023 Nov 6;52(D1):D1089–96. doi: 10.1093/nar/gkad990 (PMC10767845; doi:10.1093/nar/gkad990)
Supplement: gkad990_Supplemental_Files [file gkad990_supplemental_files.zip › SupplementaryTable_legends.pdf]

Supplementary Table 1:
Comparable single-cell RNA sequencing databases with their total number of sampled brain cells and included studies.

Supplementary Table 2:
The complete listing of single-cell RNA sequencing studies included in the ZEBRA database, sorted by the number of raw-cells.
